# Echocardiographic Right Ventricular Wall Tension Indicates Disease Severity in Children With Pulmonary Arterial Hypertension

**DOI:** 10.1016/j.jacadv.2022.100055

**Published:** 2022-08-26

**Authors:** Hosan Hasan, Philippe Chouvarine, Georg Hansmann

Precapillary pulmonary hypertension (PH) caused by pulmonary vascular disease results in hypertrophy, dysfunction, and dilation of the right ventricle (RV), and ultimately right heart failure preceding death or lung transplantation. Survival in pediatric and adult PH cases has improved over the last 2 decades because of earlier and more accurate diagnosis, better risk stratification, and earlier initiation of dual/triple pharmacotherapy. Nevertheless, transplant-free survival in patients with idiopathic pulmonary arterial hypertension, heritable pulmonary arterial hypertension, and other forms of World Health Organization group 1 PH remains poor.

To address the unmet need of better risk stratification in children with PH, the European Pediatric Pulmonary Vascular Disease Network (EPPVDN) developed^1^ and started to validate a new pediatric PH risk score in a prospective add-on selexipag study^2^; the EPPVDN risk score combines: 1) clinical and blood (N-terminal prohormone of brain natriuretic peptide) markers; 2) echocardiographic variables, ie, qualitative right atrium/RV enlargement, qualitative RV dysfunction, RV/left ventricular (LV) end-systolic diameter ratio, tricuspid annular plane systolic excursion (TAPSE), pulmonary artery acceleration time, systolic/diastolic duration ratio; and 3) (optionally) invasive hemodynamic data, to generate decimal numbers that indicate lower and higher PH risk of clinical deterioration.

**Figure 1:**
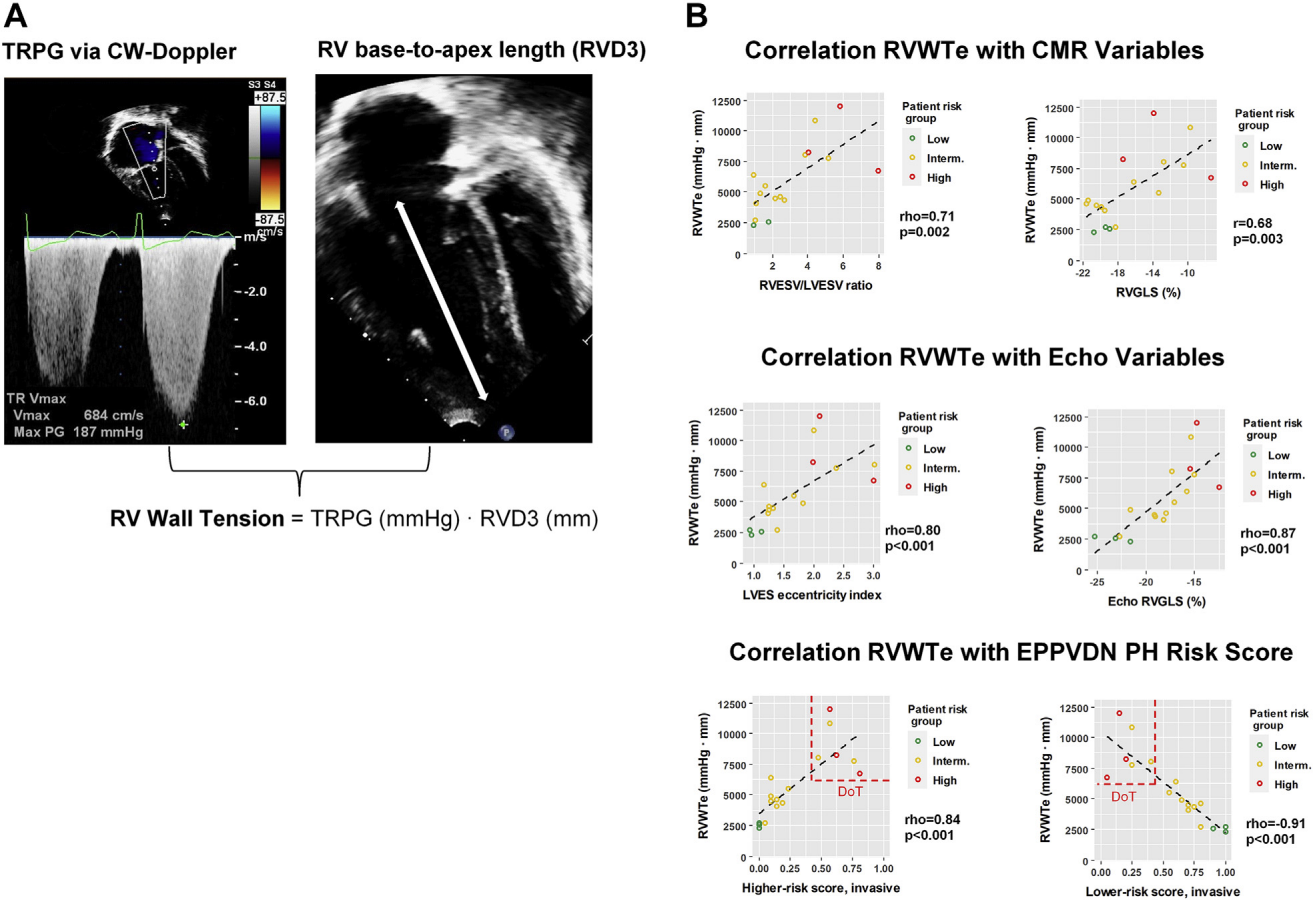
**RVWTe Correlates Significantly With CMR and Echo Imaging Variables and With the EPPVDN PH Risk Score** The simplified RVWTe was calculated according to the Laplace-Young’s law by multiplying TRPG in mm Hg with RV base-to-apex length **(white arrow)** in mm **(A)**. RV anterior wall diameter was not taken into account because of the subjectivity/variability of such measurements. Echocardiographic images from an end-stage PAH patient 3 months before lung transplantation are shown. Correlations of CMR variables, echo variables, and EPPVDN PH risk score versions with RVWTe are visualized by scatter plots with added regression fit lines (regardless of whether Pearson’s r or Spearman’s rho was calculated) **(B)**. Six of the 17 PAH patients subsequently died (n = 1) or underwent lung transplantation (n = 5); those 6 death-or-transplantation (DoT) patients are shown inside the dashed rectangles (highest RVWTe, worst risk scores). Pearson’s r or Spearman’s rho correlation coefficients are used based on normality test (Shapiro-Wilk). Scatter plots were created using R-software (ver.4.0.5). CMR = cardiac magnetic resonance; EPPVDN = European Pediatric Pulmonary Vascular Disease Network; PAH = pulmonary arterial hypertension; PH = pulmonary hypertension; RV = right ventricle; RVGLS = RV global longitudinal strain; RVWTe = right ventricular wall tension; TRPG = tricuspid regurgitation pressure gradient.

Recently, simplified echocardiographic RV wall tension (RVWTe), defined as the product of tricuspid regurgitation (TR) pressure gradient (TRPG) (Figure 1A) and end-diastolic base-to-apex length (Figure 1B), was identified as an echocardiographic predictor of altered invasive RV hemodynamics in adults with PH^3^: RVWTe was increased early in the disease process and associated with all-cause mortality at ∼5-year follow-up, while TAPSE, RV fractional area change, TRPG (calculated from TR velocity), and TAPSE/TR velocity ratio were not.^3^

In a separate, retrospective study of 1,142 adult patients with moderate-to-severe secondary TR (all causes, congenital heart disease excluded) and ∼4.5-year mortality of 51%, increased RVWTe was independently associated with worse prognosis at a cutoff of >3,300 mm Hg × mm (∼4.5-year mortality HR: 1.555; 95% CI: 1.268-1.907; *P* < 0.001).^4^ However, so far, RVWTe has not been studied in children with PH.

In the current prospective, exploratory study (institutional review board #2200), children with pulmonary arterial hypertension (PAH) underwent transthoracic echocardiography, cardiac magnetic resonance (CMR), and combined right-left heart catheterization, to calculate noninvasive and combined noninvasive/invasive PH risk scores.^1^ Patients without a reliable TR-Doppler envelope were excluded. As expected, healthy children did not have sufficient TR envelopes to calculate normal pediatric reference values for RVWTe. All imaging studies were performed during the same inpatient stay. CMR examination was pursued at median intervals of 2 days apart from cardiac catheterization and echocardiography (range: 1-6 days). The physician analyzing the echocardiographic images was blinded for CMR and EPPVDN risk score values. The demographic data of the pediatric PAH cohort (n = 17) included age (4.0-17.6 years), female sex (9/17), body weight (14-65 kg), and body surface area (0.65-1.77 m^2^). The disease severity range was broad (serum N-terminal prohormone of brain natriuretic peptide <50-4,436 ng/L; World Health Organization functional class 1-3), and PH subgroups included 6 idiopathic pulmonary arterial hypertension, 6 heritable pulmonary arterial hypertension, 2 PAH-congenital heart disease, 2 PAH-interstitial lung disease, and 1 PAH-pulmonary veno-occlusive disease. We tested interobserver variability with respect to RVTWe using 2 independent measurements and found it to be extremely low (2-way, agreement, single-score intraclass correlation coefficient = 0.997; calculated using the irr R package [ver.0.84.1]).

We found the echocardiographic RVWTe (=TRPG × end-diastolic base-to-apex length) to be abnormally increased in pediatric PAH (median: 4,867 [IQR: 4,100-7,768], range: 2,265-11,985 mm Hg × mm; n = 17) vs the adult cutoff for increased mortality with secondary TR (3,300 mm Hg × mm)^4^ and to correlate with clinically relevant CMR-derived variables; the latter included RV/LV end-systolic volume ratio and RV global longitudinal strain (Figure 1B) as well as RV/LV end-diastolic volume ratio (rho = 0.69, *P* = 0.003) and RV ejection fraction (r = −0.53, *P* = 0.034), all of which have been shown to prognosticate mortality in adult PAH patients. In our pediatric PAH cohort, RVWTe also correlated strongly (rho or r ≥ 0.8) with echocardiographic measures of RV contractility, dilation and interventricular septal shift: RV global-longitudinal strain and LV end-systolic eccentricity index (Figure 1B) as well as RV/LV end-systolic diameter ratio (rho = 0.87, *P* < 0.001) and RV free wall longitudinal strain (r = 0.65, *P* = 0.005).

Intriguingly, RVWTe correlated very strongly with the EPPVDN risk scores (Figure 1B). Along the RVWTe, the pediatric PAH patients clustered into those at lower (green), intermediate (yellow), or higher (red) risk of clinical deterioration (Figure 1B). Six of the 17 PAH patients subsequently underwent bilateral lung transplantation or died (n = 1), and those 6 death-or-transplantation patients had (non)invasive higher risk scores >0.5 (mean [standard deviation]: 0.69 [0.07]; range: 0.53-0.93) and very high RVWTe values ≥6,740 mm Hg × mm (8,936 ± 827; range: 6,740-11,985 mm Hg × mm), indicating end-stage PAH.^5^

To the best of our knowledge, this report is the first to demonstrate increased RVWTe in children with PAH and to validate the RVWTe by correlating RVWTe with CMR-derived variables known to prognosticate clinical outcomes. Most importantly, despite the small cohort, the simplified RVWTe correlates very strongly with the new EPPVDN PH risk score. Thus, RVWTe has the potential to become an easily applicable, prognostic echocardiographic marker of PH severity in pediatric PH patients with a reliable TRPG-Doppler measurement. Nevertheless, further work in larger patient cohorts is needed to determine the feasibility and reproducibility of RVWTe as a reliable imaging marker for risk assessment in pediatric PH.
